# Supplementary material for: Generalized bacterial genome editing using mobile group II introns and Cre-lox
Source: Mol Syst Biol. 2013 Sep 3;9:685. doi: 10.1038/msb.2013.41 (PMC3792343; doi:10.1038/msb.2013.41)
Supplement: Supplementary Information — Supplementary Figures S1-5, Supplementary Tables S1 and 2 [file msb201341-s1.pdf]

Supplementary Information

Generalized bacterial genome editing using mobile group II introns and Cre-*lox*

Peter J. Enyeart[1], Steven M. Chirieleison[2,5], Mai N. Dao[3,4], Jiri Perutka[1,3], Erik M. Quandt[1], Jun Yao[1,3,4], Jacob T. Whitt[1,3,4], Adrian T. Keatinge-Clay[1,3], Alan M. Lambowitz[1,3,4] & Andrew D. Ellington[1,3,6]

[1]Institute for Cell and Molecular Biology, University of Texas at Austin, Austin, Texas, 78712 USA.
[2] Department of Biomedical Engineering, University of Texas at Austin, Austin, Texas, 78712 USA.
[3]Department of Chemistry and Biochemistry, University of Texas at Austin, Austin, Texas, 78712 USA.
[4]Section of Molecular Genetics and Microbiology, School of Biological Sciences, University of Texas at Austin, Austin, Texas, 78712 USA.
[5]Current address: School of Medicine, Case Western Reserve University, Cleveland, Ohio, 44106 USA.
[6]Corresponding author. Email andy.ellington@mail.utexas.edu.

Contents:

Supplementary Text
Supplementary Methods
Supplementary Figures 1 through 5
Supplementary Tables 1 and 2

Supplementary Text

Detailed discussion of statistical analyses. Statistical analyses were performed in R. For the data on the dependency of intron efficiency on insert type in **Figure 2**, analyses were performed on square-root-transformed data in order to obtain better homoscedasticity, which is a requirement for analysis of variance (ANOVA) comparisons (Rosner, 2011). Barlett's test for equality of variances (Rosner, 2011) gave 8.1×10^{-5} for the untransformed Ll.LtrB values, 0.30 for the transformed Ll.LtrB values, 0.10 for the untransformed EcI5 values, and 0.77 for the transformed EcI5 values. For normality tests, all data triplets gave a *P*-value of at least 0.01 in the Shapiro-Wilk normality test (Shapiro & Wilk, 1965), except the values for the 1WL1 insert in Ll.LtrB, which contained two values of zero. Replacing the LtrB.1WL1 data with values randomly selected from a normal distribution having the same mean as the actual data points (0.084%) and a standard deviation equal to 0.08% made no substantial difference in the results. Multiple pairwise comparisons for this and all other data were made using the Tukey method for correcting for multiple testing (Hsu, 1994).

An analysis of variance (ANOVA) of the results presented in **Figure 2** confirmed the dependence of intron efficiency on insert type (with *P*-values of 2.93×10^{-9} for LtrB.LacZ.635s and 3.04×10^{-11} for EcI5.LacZ.912s). The pairwise comparisons also confirmed that the inserts generally fall into two groups: one of approximately wild-type efficiency (with flexible structures) and one of markedly impaired efficiency (with relatively rigid structures). The *P*-values for the difference between the least efficient insert in the wild-type group and the most efficient insert in the impaired group were 6.8×10^{-5} for Ll.LtrB and 6.0×10^{-6} for EcI5.

The analyses of the data on Cre-mediated insertion (RMCE) efficiency were also performed on square-root-transformed values. The *P*-values of Bartlett's test for transformed and untransformed data were 0.11 and 0.99, respectively, for the data shown in **Figure 5B**, and 4.4×10^{-5} and 0.37, respectively, for the data shown in **Figure 5C**. Even for the data shown in **Figure 5B**, the *P*-values resulting from an ANOVA performed on the square-root-transformed data were more stringent and were in better agreement with the results of pairwise comparisons, and thus the square-root-transformed data was used for the analysis. All triplets had *P*-values of at least 0.04 in the Shapiro-Wilk normality test, except for HMS174(DE3) containing a high-copy vector on day three, where all three values were 100%. Replacing these values with values randomly drawn from a normal distribution having a mean of 100% and a standard deviation of 1% (similar to the other triplets near 100%) made no substantial difference in the analysis results.

A multifactorial analysis of variance performed on the results in **Figure 5B** indicated that time (*P*-value = 7.837×10^{-14}) and delivery-plasmid copy number (*P*-value = 2.363×10^{-8}) were significant factors, but strain (*P*-value = 0.2708) was not. However, the interactions between strain and time (*P*-value = 1.588×10^{-5}) and between strain and copy number (*P*-value = 7.243×10^{-7}) were significant. The interaction between copy number and time (*P*-value = 0.04234) and

the three-way interaction between all factors (*P*-value = 0.02650) were significant at the 0.05 level but not the 0.01 level. Subsequent comparisons between days (corrected for multiple comparisons) showed a significant difference between day one and day two (*P*-value = 1.1×10^{-5}) and between day one and day three (*P*-value = 1.0×10^{-6}), but not between day two and day three (*P*-value = 0.698).

A multifactorial analysis of variance performed on the results in **Figure 5C** indicated that location significantly affects insertion efficiency (*P*-value = 7.2×10^{-7}). Time also proved once again to be a significant factor, with a *P*-value of 2.6×10^{-13}. Subsequent pairwise comparisons, corrected for multiple testing, showed that the *malT* locus differed significantly from the *lacZ* and *galK* loci on days two and three (maximum *P*-value = 4.1×10^{-3}) but not on day one (minimum *P*-value = 0.61). The *lacZ* and *galK* loci were not found to differ significantly (*P*-value = 0.72).

For the doubling-time data in **Table 2**, all strains had a Shapiro-Wilk *P*-value of at least 0.01. Bartlett's test gave a *P*-value of 0.13 for the original data and 0.19 for square-root-transformed data. Using square-root-transformed data versus untransformed data made little difference in the quantitative results (*P*-values) and no difference in qualitative results (determinations of statistically significant differences), and thus the *P*-values presented below for doubling times are those for untransformed data.

An analysis of variance performed on the results in **Table 2** showed that doubling time is highly dependent on the type of rearrangement present (*P*-value = 1.85×10^{-14}). Subsequent pairwise comparisons corrected for multiple testing showed that the strains fall broadly into groups: a high-growth group having approximately wild-type doubling times and a low-growth group having impaired doubling times, where the *P*-value for the difference between the slowest member of the high-growth group and the fastest member of the low-growth group was 0.028.

Supplementary Methods

Plasmid construction

Plasmids used in the present work are listed in **Supplementary Table 3**. Introns were retargeted as described in the **Methods** section of the main text. To insert *lox* constructs into the introns, the intron plasmids were first cut with MluI in the presence of calf intestinal phosphatase. The *lox* inserts themselves were ordered as two complementary oligomers (oligomers are listed in **Supplementary Table 5**) that were annealed together by mixing 10 μL of 200-μM solutions of each of the oligomers with 80 μL of water, holding at 95°C for 20 minutes, and then allowing to cool (in some cases by ramping downward at 0.5°C/s until reaching 40°C, holding 20 minutes at 40°C, and then cooling at room temperature). The annealed oligomers were then ligated directly into the MluI-cut intron plasmids.

The pACD3 plasmid was used as the starting point for gene delivery plasmids. pACD3 was cut with AvaI and HindIII to remove the *ltr* operon, leaving the T7 promoter intact. The resulting vector backbone and the annealed 2ML6 oligomers (see **Supplementary Table 5**) were then blunted using Klenow fragment and ligated together to give the plasmid pACDX3. This vector was then amplified using the primers vlu and vld, and the *sacb* gene was amplified from the plasmid pPSBA2KS (Lagarde et al, 2000) using the primers sul and sdl2. These two PCR products were then ligated together using the PIPE method (Klock et al, 2008), giving plasmid pACDX3S, which has the *sacB* gene inserted in place of the T7 promoter in pACDX3.

Assembling components for GFP insertion. To generate the plasmid pUC19X3S-GFPb, which contains a promoterless GFP ORF appended with terminators at the 3' end inserted into the PmeI site of the 2ML6R oligomer sequence, the *sacB* gene and lox sites were first amplified from pACDX3S using the primers sac.2ml6.F and sac.2ml6.R. The resulting PCR product was digested with SacI and BamHI and inserted into the multiple cloning site of pUC19 in place of the SacI and BamHI fragment, generating plasmid pUC19X3S. Oligomers mcs.F and mcs.R were annealed and cloned into the PmeI site within 2ML6 to add a multiple cloning site, generating pUC19X3Sm. The GFPuv open reading frame was PCR amplified from pGFPuv (Clontech) in sequential PCR reactions with primers at.rbs.gfp.f and gfp.term.r followed by eagi.gfp.f and spei.gfp.r and cloned after digestion into the EagI and SpeI site of pUC19X3Sm, yielding pUC19X3S-GFPa. A second T7 terminator was then inserted by ligating oligomers spei.term.f and spei.term.r into the SpeI site of pUC19X3S-GFPa, generating pUC19X3S-GFPb. The pUC19X3S-GFPb plasmid carries the GFPuv open reading frame with no promoter followed by two T7 terminators and is flanked by the *lox* sites of oligomer 2ML6R.

Generating GFP insertion plasmids. The pUC19X3S-GFPb plasmid was used as PCR template to generate the GFP donor plasmids pUC19X3S-GFP and pACDX3S-GFP. To generate plasmid pUC19X3S-GFP, the GFP construct with flanking lox sites was amplified from pUC19X3S-

GFPb in three consecutive PCR reactions, using primers gfp.t1.f and gfp.r, gfp.t2.f and gfp.r, and finally gfp.t3.f and gfp.spei.r. These three sequential PCR reactions were performed to append T7 terminators upstream of the 5' lox sites to prevent GFP expression without Cre-mediated recombination. The backbone of plasmid pUC19X3S-GFPb was amplified using primers puc19x3s.spei and noti.r. After digestion with SpeI and NotI, the pUC19X3S-GFPb backbone and the final PCR for the GFP insertion construct were ligated, generating plasmid pUC19X3S-GFP.

The pACDX3S-GFP donor plasmid was generated from pACDX3S. The GFP insertion construct was amplified from pUC19X3S-GFPb in three consecutive PCR reactions, using primers gfp.t1.f and gfp.r, gfp.t2.f and gfp.r, and finally gfp.t3.f and gfp.sphi.r. The backbone of plasmid pACDX3S was amplified using primers pacdx3s.sphi and noti.r. After digestion with SphI and NotI, the pACDX3S backbone and the final PCR were ligated to generate plasmid pACDX3S-GFP. Plasmids pACDX3S-GFP and pUC19X3S-GFP were used in GFP insertion assays to screen efficiency of CRE-mediated insertion to the genome.

*Introns for delivering T7 promoter to genome***.** Plasmid pACD.EcI5.LacZ.1806s.T7s.2ML5R was initially used to deliver a T7 promoter to the genome for subsequent Cre-mediated insertion efficiency screens with promoterless GFP delivery plasmids. This plasmid expresses the EcI5 intron containing a T7 promoter oriented such that transcription proceeds in the direction of the 2ML5R lox sites. First, plasmid pACD.EcI5.LacZ.1806s was digested with MluI, dephosphorylated, and ligated with annealed oligomers T7s and T7as, yielding plasmid pACD.EcI5.LacZ.1806s.T7s, with a single MluI downstream of the T7 promoter. To insert the lox sites, this plasmid was digested with MluI at the conserved site, dephosphorylated, and ligated with annealed oligomers 2ML5F and 2ML5R, yielding insertion of the 2ML5R sequence at the MluI site and generating plasmid pACD.EcI5.LacZ.1806s.T7s.2ML5R. pACD.EcI5.GalK.433s.T7s.2ML5R and pACD.EcI5.MalT.739a.T7s.2ML5R, which were used to deliver the T7 promoter and lox sites to the *galK* and *malT* loci, were constructed in the same manner.

Generating the polyketide synthase insertion donor plasmid. pET26b-DEBS1TE was constructed in the Keatinge-Clay lab by ligating the SacI-EcoRI fragment (TE) obtained from pKOS422-100-1 (Mod2TE; (Menzella et al, 2005)) into the equivalent sites of pKOS422-33-1 (DEBS1; (Menzella et al, 2006)). The pET26b-DEBS1TE plasmid was used to generate the polyketide synthase insertion plasmid, pET26b-DEBS1TE-i. To begin, plasmid pET26b-DEBS1TE was digested with NotI and ligated with annealed oligomers term.lox66.f and term.lox66.r, yielding an insertion of term.lox66.f sequence with a single NotI site at the 5' end (plasmid pET26b-DEBS1TE-tP). Plasmid pET26b-DEBS1TE-tP was digested with NotI and ligated with annealed oligomers stop.term.f and stop.term.r, generating plasmid pET26b-DEBS1TE-sttP with the stop.term.f sequence inserted to the NotI site. Plasmid pET26b-DEBS1TE-sttp was digested with EcoRV and ligated with annealed oligomers loxm271.f and loxm271.r, yielding the plasmid

pET26b-DEBS1TE-i with the loxm271.f sequence at the EcoRV site. The pET26b-DEBS1TE-i insertion plasmid contains the DEBS1-TE polyketide synthase module flanked by loxm2/71 upstream of the T7 promoter and lox66 downstream from the transcription terminators.

Construction of pX10, pX11, pX20, and pX21. The plasmid pACDX2S was constructed in the same manner as pACDX3S above, except annealed 2ML2 oligomers (see see **Supplementary Table 5**) were used instead of the 2ML6 oligomers. A pair of T7 terminators was amplified from the pUC19XS.GFP plasmid using primers term3f and term3r. These were ligated into XbaI-cut and Klenow-blunted pACDX2S and pACDX3S to yield pX10 and pX20, respectively. The oligomers pucmcs and pucmcsr were then annealed together and then ligated into PmeI-cut pX10 and pX20 to yield pX11 and pX21, respectively.

S. oneidensis 16s rDNA intron. Plasmid RP4.T5.rDNA.798s was generated in a 3-step cloning procedure. First the 798s intron was cloned into plasmid pACD3 by digestion of pACD3 and 798s.gBLOCK (IDT) targeting region with BsrGI and HindIII. Vector and insert fragments were purified and ligated to produce plasmid pACD3.rDNA.798s.

The rDNA.798s intron was then PCR amplified from plasmid pACD3.rDNA.798s with primers T5.LtrB.pBAV.1 and T5.LtrB.pBAV.2 and inserted into plasmid pBAV1k -lacI-PT5-gusA (Murin et al, 2012) using overlap extension PCR cloning (Bryksin & Matsumura, 2010) to create plasmid pBAV1K.lacI.T5.rDNA.798s. Plasmid pBAV1K.lacI.T5.rDNA.798s was then PCR amplified with primers RP4.T5.pBAV.F and RP4.pBAV.R and the resulting T5.rDNA.798s fragment was cloned into plasmid RP4.MCS digested with XbaI by Gibson isothermal assembly (Gibson et al, 2009) to create plasmid RP4.T5.rDNA.798s.

Plasmid RP4.MCS was created by inserting a multiple cloning site containing: AscI, SpeI, XbaI, NheI, PmeI, PacI and SacI restriction sites into the EcoRI site of plasmid RP4. This was performed by PCR amplifying an oligonucleotide containing the MCS (RP4.MCSseq) with primers MCS.RP4.F and MCS.RP4.R and cloning this fragment via Gibson isothermal assembly(Gibson et al, 2009) into plasmid RP4 digested with EcoRI.

Supplementary References

Bryksin AV, Matsumura I (2010) Overlap extension PCR cloning: a simple and reliable way to create recombinant plasmids. *Biotechniques* **48:** 463-465

Gibson DG, Young L, Chuang RY, Venter JC, Hutchison CA, 3rd, Smith HO (2009) Enzymatic assembly of DNA molecules up to several hundred kilobases. *Nature Methods* **6:** 343-345

Hsu JC (1994) *Multiple Comparisons: Theory and Methods*: Chapman & Hall.

Klock HE, Koesema EJ, Knuth MW, Lesley SA (2008) Combining the polymerase incomplete primer extension method for cloning and mutagenesis with microscreening to accelerate structural genomics efforts. *Proteins* **71:** 982-994

Lagarde D, Beuf L, Vermaas W (2000) Increased production of zeaxanthin and other pigments by application of genetic engineering techniques to Synechocystis sp. strain PCC 6803. *Appl Environ Microbiol* **66:** 64-72

Menzella HG, Reid R, Carney JR, Chandran SS, Reisinger SJ, Patel KG, Hopwood DA, Santi DV (2005) Combinatorial polyketide biosynthesis by de novo design and rearrangement of modular polyketide synthase genes. *Nat Biotechnol* **23:** 1171-1176

Menzella HG, Reisinger SJ, Welch M, Kealey JT, Kennedy J, Reid R, Tran CQ, Santi DV (2006) Redesign, synthesis and functional expression of the 6-deoxyerythronolide B polyketide synthase gene cluster. *J Ind Microbiol Biotechnol* **33:** 22-28

Murin CD, Segal K, Bryksin A, Matsumura I (2012) Expression vectors for Acinetobacter baylyi ADP1. *Appl Environ Microbiol* **78:** 280-283

Rosner B (2011) *Fundamentals of biostatistics*, 7th edn. Boston: Brooks/Cole, Cengage Learning.

Shapiro SS, Wilk MB (1965) An Analysis of Variance Test for Normality (Complete Samples). *Biometrika* **52:** 591-&

Supplementary Figures

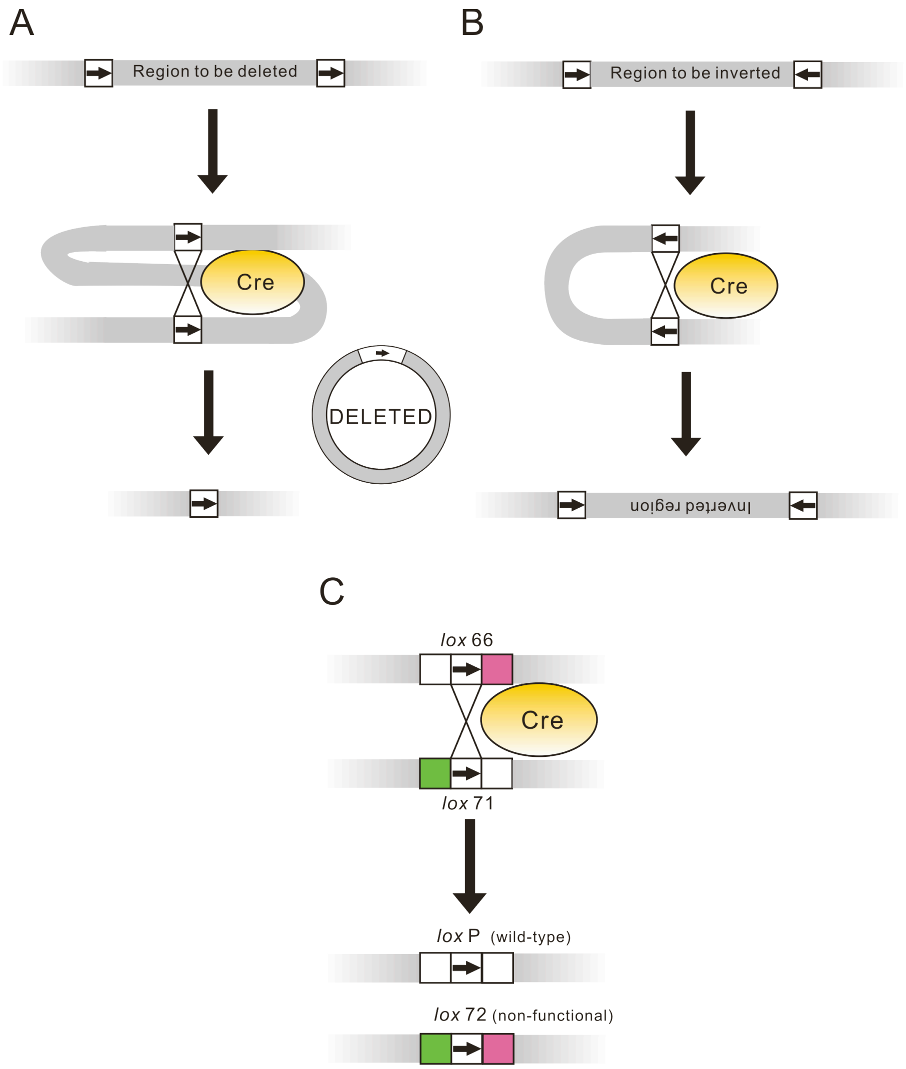

Supplementary Figure 1. Fundamentals of Cre/*lox* interactions. **(A)** When the two *lox* sites have compatible linkers with identical orientations, Cre-mediated recombination leads to a deletion. **(B)** When the two *lox* sites have compatible linkers with opposite orientations, Cre-mediated recombination leads to an inversion. **(C)** The use of *lox* sites with mutations in the palindromic arms allows unidirectional recombinations to be performed. Upon recombination between a *lox*71 and a *lox*66 site, a (wild-type) *lox*P and a *lox*72 site result. The *lox*71 and *lox*66 sites are recognized by Cre, but the *lox*72 site is not, and its formation therefore prevents recombination back to the original state. In the figure, *lox* sites are represented by three boxes (arm, linker, arm), where white represents wild-type *lox*P sequence, green represents the *lox*71 mutant arm, pink represents the *lox*66 mutant arm, and the arrows represent the linker orientation.

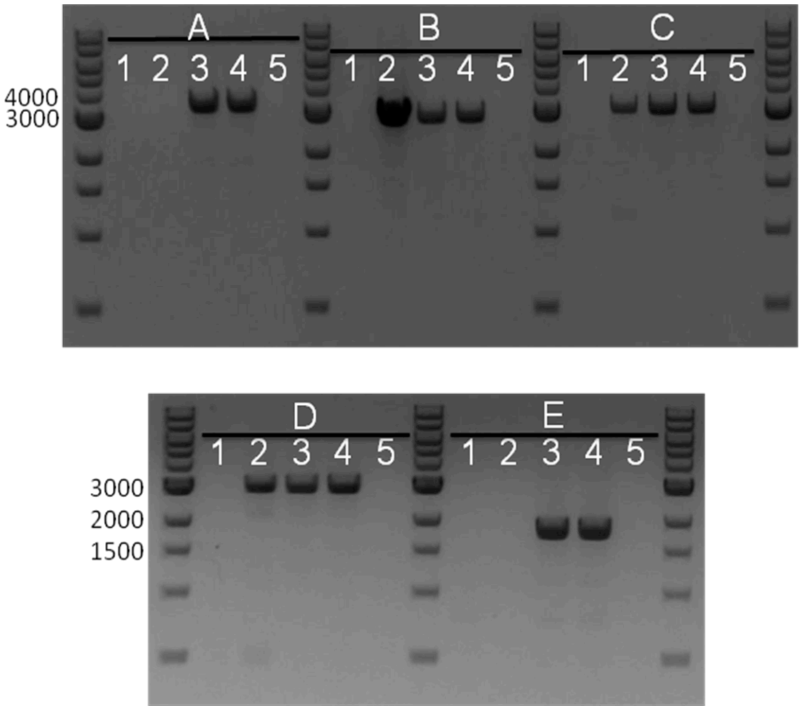

Supplementary Figure 2. Verification of DEBS1-TE (polyketide synthase operon) genomic insertion. A through E are overlapping PCRs covering the entire 12-kb operon, where the A and E PCRs in particular also amplify the flanking DNA intron sequence in the genome and should only be seen upon successful insertion. Lane 1: Unmodified *E. coli* K207-3; Lane 2: Plasmid pET26b-DEBS1TE-i (DEBS1-TE delivery plasmid); Lane 3: DEBS1-TE insertion clone 1 (*E. coli* K207-3 base strain); Lane 4: DEBS1TE insertion clone 2 (*E. coli* K207-3 base strain); Lane 5: Negative control (water). All bands are of the expected sizes and were further verified by sequencing. The primers used are listed in **Supplementary Table 5**.

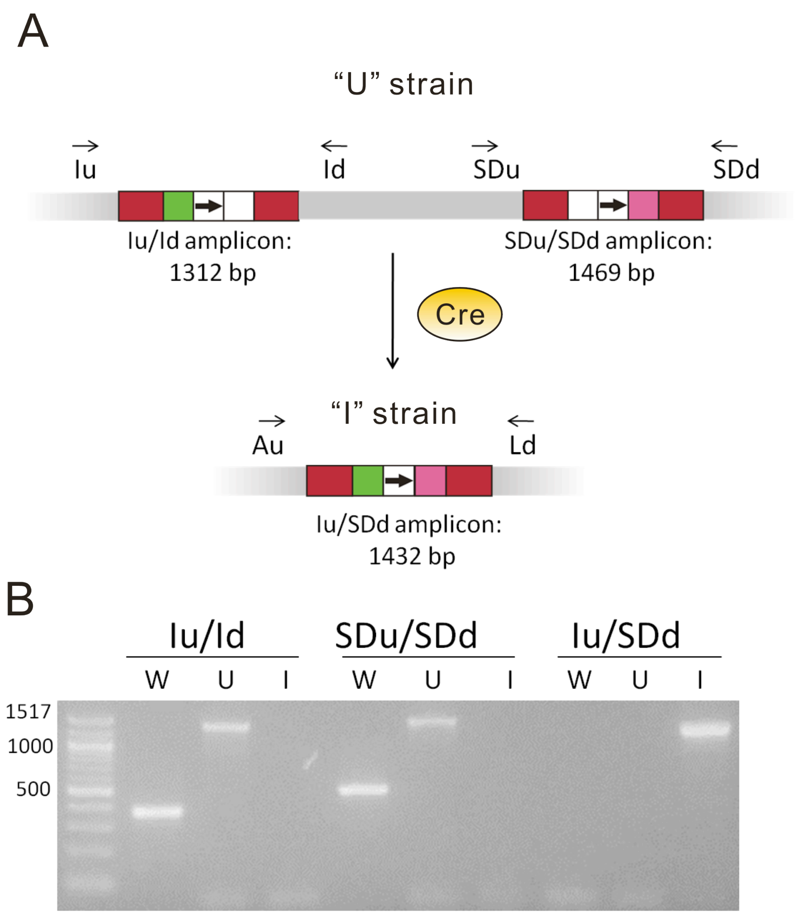

Supplementary Figure 3. Deletion in *Staphylococcus aureus*. Letter designations are as described in **Figure 6**. **(A)** Methodology, showing schematics of the PCRs used to verify the deletions, where Iu and Id primers amplify the *int* insertion site, and the SDu and SDd primers amplify the *SAPI-B* insertion site. **(B)** Verification of the strain (*S.aureus* RN10628 E1) containing a deletion of the SaPI (*int*/*SAPI-B*) region, as shown in (A). The Iu/SDd-I band was further verified by sequencing.

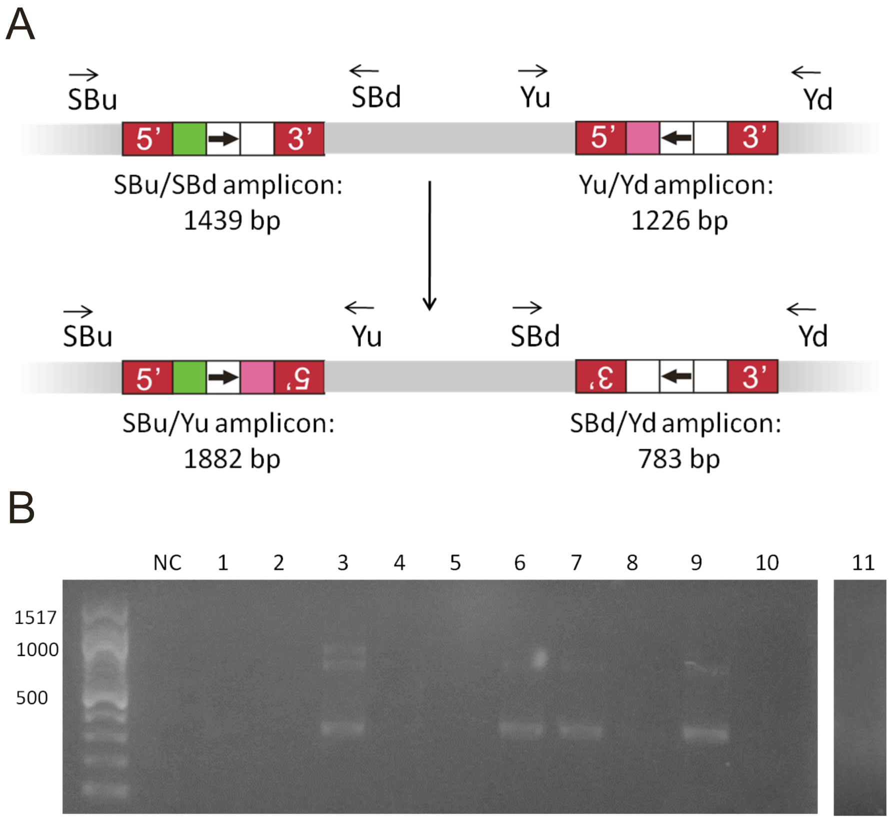

Supplementary Figure 4. Inversion in *Bacillus subtilis*. Letter designations are as described in **Figure 6**. **(A)** Methodology, where the SBu and SBd primers amplify the *sacB* intron insertion site, and the Yu and Yd primers amplify the *yhcS* insertion site. **(B)** Screening for inversions via PCR using the SBd/Yd primer pair on *B. subtilis* colonies containing intron insertions as depicted at the top in (A), after the addition of the Cre-expressing plasmid. The negative control (NC) was the same strain, except without the addition of Cre. The smaller, brighter bands are consistent with deletion of the inverted repeat formed by the inversion, but dimmer bands corresponding to the expected amplicon size are seen in all four lanes that gave bands. (The source of the uppermost band in lane 3, but it is assumed to be an artifact.) All four PCR products were sequenced, and the results confirmed the occurrence of the expected inversion between the *sacB* and *yhcS* loci followed by removal of the intron and *lox* sequences by homologous recombination. These bands were not found in individual colonies upon restreaking, and thus the inversion was judged to be unstable.

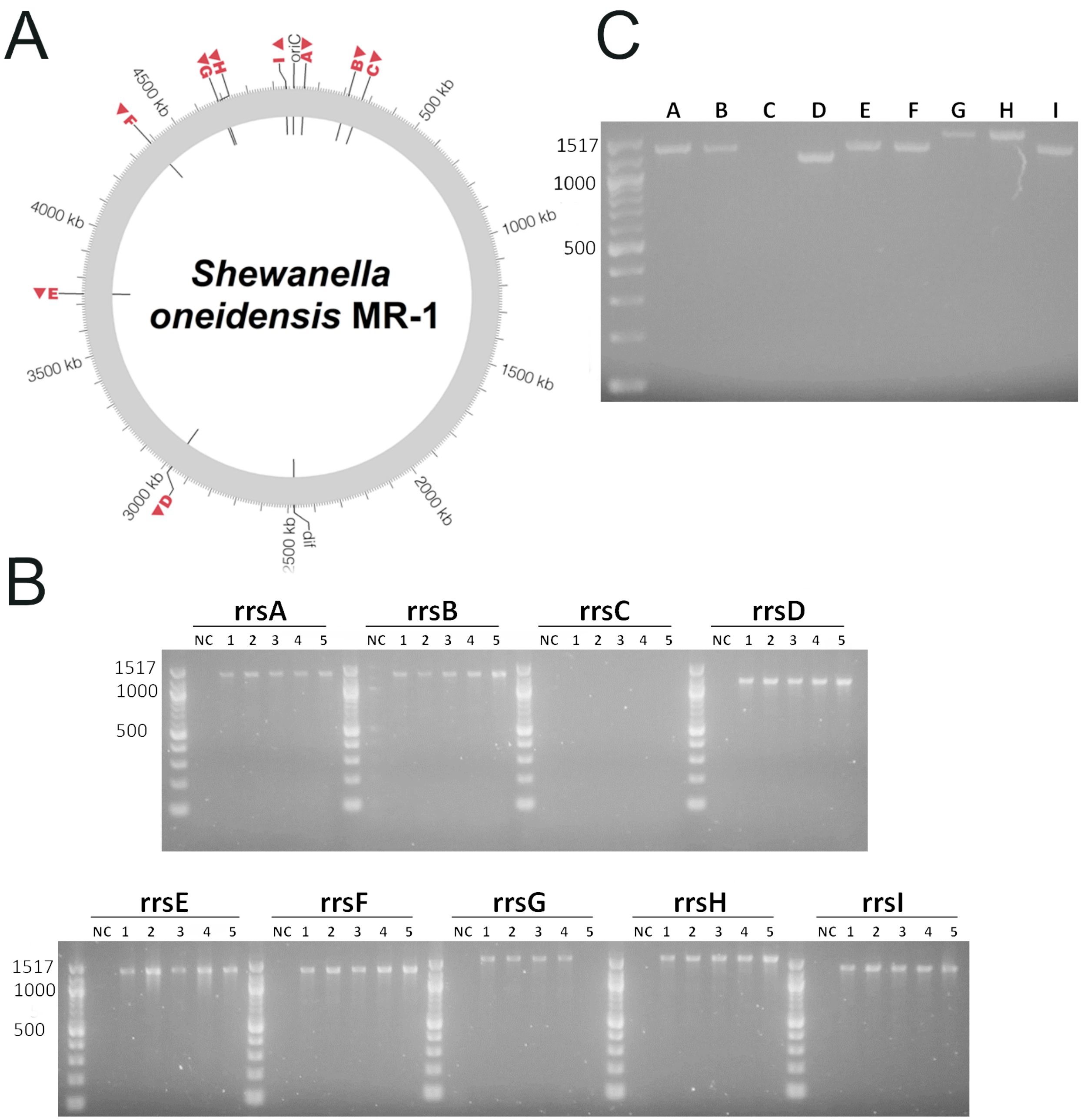

Supplementary Figure 5. Modifications in *Shewanella oneidensis*. **(A)** Schematic of the *S. oneidensis* genome, showing locations and orientations of the *rrs* genes. **(B)** The results of PCR amplifications to determine intron insertions into each *rrs* gene in five isolates of *S. oneidensis* transformed with RP4.T5.rDNA.798s.1WL2R. One primer binds to intron sequence, and the other binds to a unique genomic region outside the *rrs* gene. The "NC" lanes were performed on untransformed cells. **(C)** A repetition of the PCRs in (B) performed on a single colony grown by inoculating isolate 5 into liquid culture and streaking the overnight culture on a plate.

Supplementary Table 1. Introns used in the present work.

LtrB.LacZ.635s (*E. coli*; insertion site sequence from strain MG1655)
Source: Perutka & Lambowitz, unpublished results.

```
LtrB IS          -30       -25       -20       -15       -10       -5      -1+1      +5        +10       +15  Score:
364894|364895(-)  T A T G T G G C G G A T G A G C G G C A T T T T C C G T G A C G T C T C G T T G C T G C A   8.57
```

Inserts in sense strand of *lacZ*.
Insertion efficiency*: 15.3%

Retargeting primers (constant primer listed in **Supplementary Table 5**)†:

```
635s-IBS      AAAAAAGCTTCGTCGATCGTGAACATTTTCCGTGAGTGCGCCCAGATAGGGTG
635s-EBS1     CAGATTGTACAAATGTGGTGATAACAGATAAGTCCCGTGACGTAACTTACCTTTCTTTGT
635s-EBS2     TGAACGCAAGTTTCTAATTTCGGTTAAATGTCGATAGAGGAAAGTGTCT
```

EcI5.LacZ.912s (*E. coli*; insertion site sequence from strain MG1655)
Source: Zhuang et al., 2009

```
EcI5 IS            -30       -25       -20       -15       -10       -5      -1+1      +5        +10       +15  Score:
364617|364618(-)    A A C G T C G A A A A C C C G A A A C T G T G G A G C G C C G A A A T C C C G A A T C T C    10.62
```

Inserts in sense strand of *lacZ*.
Insertion efficiency*: 68±7.1%

Retargeting primers (constant primer listed in **Supplementary Table 5**)†:

```
912s-IBS1/2S  CCCCTCTAGAAGAATTCCCATGCCAAAACTGTGGAGCGCCGTGCGACATGAAGTCG
912s-EBS1S    CAGGCTTGAACCAAAAGGTATGTGGTTGGTTACTCCTCTGGCGCCTAGGGGTACACGGAC
912s-EBS2AS   TACCTTTTGGTTCAAGCCTGTCAGCATCTTTGGCTTGTTACTGTTAACGACGCTTCAGC
```

EcI5.LacZ.1806s (*E. coli*; insertion site sequence from strain MG1655)
Source: Zhuang et al., 2009

```
EcI5 IS            -30       -25       -20       -15       -10       -5      -1+1      +5        +10       +15  Score:
363723|363724(-)    T T T G G C G A T A C G C C G A A C G A T C G C C A G T T C T G T A T G A A C G G T C T G   11.01
```

Inserts in sense strand of *lacZ*.
Insertion efficiency*: 97±0.4%

Retargeting primers (constant primer listed in **Supplementary Table 5**)†:

```
1806s-IBS1/2S CCCCTCTAGAAGAATTCCCATGCCAAACGATCGCCAGTTCGTGCGACATGAAGTCG
1806s-EBS1S   CAGGCTTGAACCAAAAGGTATGTGGTTGGTTACTCCTCTGAACTCTAGGGGTACACGGAC
1806s-EBS2AS  TACCTTTTGGTTCAAGCCTGTCAGCATCTTTGGCTTGTTCGATCTAACGACGCTTCAGC
```

LtrB.A (*E. coli*; insertion site sequence from strain MG1655)
Source: This study

```
LtrB IS           -30       -25       -20       -15       -10       -5      -1+1      +5        +10       +15  Score
243410|243411(-)  T G A A G T G C G G A T A A A A A C A G C A A C A A T G T G A G C T T T G T T G T A A T T   9.89
```

Insertion efficiency*: 6/239 (2.5%)

Retargeting primers (constant primer listed in **Supplementary Table 5**):

```
A-IBS        AAAAAAGCTTATAATTATCCTTAAGCAACAATGTGGTGCGCCCAGATAGGGTG
A-EBS1       CAGATTGTACAAATGTGGTGATAACAGATAAGTCAATGTGAGTAACTTACCTTTCTTTGT
A-EBS2       TGAACGCAAGTTTCTAATTTCGATTTTGCTTCGATAGAGGAAAGTGTCT
```

EcI5.B (*E. coli*; insertion site sequence from strain MG1655)
Source: This study

```
EcI5 IS             -30       -25       -20       -15       -10       -5      -1+1      +5        +10       +15  Score
1399016|1399017(-)  T G C A G A C A T T G A C C G A A A G T C A G C G T T T T G G T T T A C G C A T A G C A G   10.50
```

Insertion efficiency*: 26/94 (27.7%)

Retargeting primers (constant primer listed in **Supplementary Table 5**):

```
B-IBS1/2S    CCCCTCTAGAAGAATTCCCATGCCAAAAGTCAGCGTTTTGGTGCGACATGAAGTCG
B-EBS1S      CAGGCTTGAACCAAAAGGTATGTGGTTGGTTACTCCTCTCAAAACTAGGGGTACACGGAC
B-EBS2AS     TACCTTTTGGTTCAAGCCTGTCAGCATCTTTGGCTTGTTAGTCATAACGACGCTTCAGC
```

EcI5.C (CMP) (*E. coli*; insertion site sequence from strain MG1655)
Source: This study

```
EcI5 IS               -30       -25       -20       -15       -10       -5      -1+1      +5        +10       +15  Score
1479630|1479631(+)    T G T A T T G A T G G A G C T A A T G A T G A T G A T T C A G T T A T G G T G G T C A G T   10.85
```

Insertion efficiency*: 11/31 (35.5%)

Retargeting primers (constant primer listed in **Supplementary Table 5**):

```
C-IBS1/2S    c
C-EBS1S      CAGGCTTGAACCAAAAGGTATGTGGTTGGTTACTCCTCTTGAATCTAGGGGTACACGGAC
C-EBS2AS     TACCTTTTGGTTCAAGCCTGTCAGCATCTTTGGCTTGTTTGATGTAACGACGCTTCAGC
```

EcI5.D (*E. coli*; insertion site sequence from strain MG1655)
Source: This study

```
EcI5 IS             -30       -25       -20       -15       -10       -5      -1+1      +5        +10       +15  Score
3452370|3452371(-)  T G G A T C G C A T C G C T T A A A G T C G G G G A C A A A A A A T T G C C T G T T G T G   10.55
```

Insertion efficiency*: 8/67 (11.9%)

Retargeting primers (constant primer listed in **Supplementary Table 5**):

```
D-IBS1/2S    CCCCTCTAGAAGAATTCCCATGCCAAAAGTCGGGGACAAAGTGCGACATGAAGTCG
D-EBS1S      CAGGCTTGAACCAAAAGGTATGTGGTTGGTTACTCCTCTTTTGTCTAGGGGTACACGGAC
D-EBS2AS     TACCTTTTGGTTCAAGCCTGTCAGCATCTTTGGCTTGTTAGTCGTAACGACGCTTCAGC
```

EcI5.E (*E. coli*; insertion site sequence from strain MG1655)
Source: This study

```
EcI5 IS              -30        -25        -20        -15        -10         -5       -1+1       +5         +10        +15  Score
3466270|3466271(+)  C A C A C C G T T A A A G C G A A T C A G C G T A T C G C T G G C A T A A A G C G T T T C  10.78
```

Insertion efficiency*: 53/93 (57.0%)

Retargeting primers (constant primer listed in **Supplementary Table 5**):

```
E-IBS1/2S      CCCCTCTAGAAGAATTCCCATGCCAAATCAGCGTATCGCTGTGCGACATGAAGTCG
E-EBS1S        CAGGCTTGAACCAAAAGGTATGTGGTTGGTTACTCCTCTAGCGACTAGGGGTACACGGAC
E-EBS2AS       TACCTTTTGGTTCAAGCCTGTCAGCATCTTTGGCTTGTTTCAGCTAACGACGCTTCAGC
```

LtrB.SAPI-int (*S. aureus*, insertion site sequence from strain NCTC 8325)
Source: This study

```
LtrB IS           -30        -25        -20        -15        -10         -5       -1+1       +5         +10        +15  Score
953084|953085(-)  G A T G A A A T G G A T A G T A A A G G T T A T G T A T A T C A A G T T A A C A A A G A T   9.69
```

Inserts in sense strand of the *int* gene.
Insertion efficiency*: 27/28 (96.4%)

Retargeting primers (constant primer listed in **Supplementary Table 5**):

```
SAPI-int-IBS       AAAAAAGCTTATAATTATCCTTAGGTTACGTATATGTGCGCCCAGATAGGGTG
SAPI-int-EBS1      CAGATTGTACAAATGTGGTGATAACAGATAAGTCGTATATCATAACTTACCTTTCTTTGT
SaPI-int-EBS2      TGAACGCAAGTTTCTAATTTCGATTTAACCTCGATAGAGGAAAGTGTCT
```

LtrB.SAPI-B (*S. aureus*, insertion site sequence from strain NCTC 8325)
Source: This study

```
LtrB IS           -30        -25        -20        -15        -10         -5       -1+1       +5         +10        +15  Score
968024|968025(-)  T C T T T G G T G G A T T A A T C A T T G G T A T C G T T C C A T A T T T A T T G A A A A   7.22
```

Insertion efficiency*: 23/23 (100.0%)

Retargeting primers (constant primer listed in **Supplementary Table 5**):

```
SAPI-B-IBS       AAAAAAGCTTATAATTATCCTTATTGGTCTCGTTCGTGCGCCCAGATAGGGTG
SAPI-B-EBS1      CAGATTGTACAAATGTGGTGATAACAGATAAGTCTCGTTCCATAACTTACCTTTCTTTGT
SAPI-B-EBS2      TGAACGCAAGTTTCTAATTTCGGTTACCAATCGATAGAGGAAAGTGTCT
```

LtrB.SacB.1221s (*B. subtilis*, insertion site sequence from strain 168)
Source: This study, Yao 2008

```
LtrB IS          -30       -25       -20       -15       -10       -5      -1+1      +5        +10       +15  Score
3537232|3537233(+)T T A A A A A T G G A T C T T G A T C C T A A C G A T G T A A C C T T T A C T T A C T C A     8.9
```

Inserts into the sense strand of the *sacB* gene.
Insertion efficiency*: 47/48 (97.9%)

Retargeting primers (constant primer listed in **Supplementary Table 5**):

```
1221s-IBS       AAAAAAGCTTATAATTATCCTTACCTAACGATGTAGTGCGCCCAGATAGGGTG
1221s-EBS1      CAGATTGTACAAATGTGGTGATAACAGATAAGTCGATGTAACTAACTTACCTTTCTTTGT
1221s-EBS2      TGAACGCAAGTTTCTAATTTCGGTTTTAGGTCGATAGAGGAAAGTGTCT
```

LtrB.YhcS.168s (*B. subtilis*, insertion site sequence from strain 168)
Source: This study, Whitt 2011

```
LtrB IS          -30       -25       -20       -15       -10       -5      -1+1      +5        +10       +15  Score
995184|995184(+)  A A T A G C A C A G A T C A A G C A A A G A A C A A A G C A T C A T T T A A G C C T G A G    9.1
```

Inserts into the sense strand of the *yhcS* (*srtA*) gene.
Insertion efficiency*: 91 ± 5%

Retargeting primers (constant primer listed in **Supplementary Table 5**):

```
186s-IBS        AAAAAAGCTTATAATTATCCTTAAAGAACAAAGCAGTGCGCCCAGATAGGGTG
186s-EBS1       CAGATTGTACAAATGTGGTGATAACAGATAAGTCAAAGCATCTAACTTACCTTTCTTTGT
186s-EBS2       TGAACGCAAGTTTCTAATTTCGGTTTTCTTCCGATAGAGGAAAGTGTCT
```

LtrB.rDNA.798s *(S. oneidensis*, insertion site sequence from strain MR-1 *rrsA* gene)
Source: This study

```
LtrB IS          -30       -25       -20       -15       -10       -5      -1+1      +5        +10       +15  Score
787|788s          A A G C G T G G G G A G C A A A C A G G A T T A G A T A C C C T G G T A G T C C A C G C C
```

Inserts into the sense strand of the *rrs* genes in *S. oneidensis*.
Insertion efficiency*: Not directly applicable, but all tested colonies contained the insertion in most copies of the *rrs* gene.

Retargeting primers (constant primer listed in **Supplementary Table 5**):

```
798s-IBS        AAAAAAGCTTATAATTATCCTTAGGATTCGATACCGTGCGCCCAGATAGGGTG
798s-EBS1       ACAAAGAAAGGTAAGTTAAGGGTATCGACTTATCTGTTATCACCACATTTGTACAATCTG
798s-EBS2       TGAACGCAAGTTTCTAATTTCGATTAATCCTCGATAGAGGAAAGTGTCT
```

---

* Intron efficiency for LtrB.LacZ.635s is the average from three plates used in this study (**Fig. 2**); intron efficiencies for EcI5.LacZ.912s and EcI5.LacZ.1806s are as reported in Zhuang et al, 2009; intron efficiencies for LtrB.SacB.1221s are as reported in Yao, 2008; introns efficiencies for LtrB.YhcS.187s are as reported in Whitt, 2001; and intron efficiencies for all other introns

are for all uses of the intron during the course of this study and include tests in various strains and with any *lox* inserts that form hairpins with flexible bases (in addition to tests using the unmodified intron). Partial insertions (bands corresponding to both uninserted and inserted states seen upon colony PCR) were counted as insertions.
† The primers for the *lacZ* introns were inferred from the sequences of the intron-expressing plasmids and were not used to construct the introns used in this study.

Supplementary Table 2. Expected sizes of amplicons for verifying intron insertions and Cre/*lox* recombinations.

Primers	Event detected	Expected size (bp)	Inverted repeat generated*	Relevant figures
Au/Ad	Ll.LtrB insertion	379 (before)<br>1335 (after)	No	6B, 6C, 7B, 8A, 8B
Bu/Bd	EcI5 insertion	367 (before)<br>1286 (after)	No	6D
Cu/Cd	EcI5 insertion	388 (before)<br>1307 (after)	No	6D
Du/Dd	EcI5 insertion	285 (before)<br>1204 (after)	No	6C, 7C
Eu/Ed	EcI5 insertion	284 (before)<br>1203 (after)	No	6C, 7C, 7D, 8A, 8B
Lu/Ld	EcI5 insertion	222 (before)<br>1141 (after)	No	6B, 6C, 7B, 7D, 8A, 8B
Lu_0/Ld_0	Ll.LtrB insertion	246 (before)<br>1226 (after)	No	7D
Iu/Id	Ll.LtrB insertion	357 bp (before)<br>1312 (after)	No	Supp. 3B
SDu/SDd	Ll.LtrB insertion	514 (before)<br>1469 (after)	No	Supp. 3B
Ad/Ld	Inversion	1747	No	7B
Au/Ld	Deletion	1308	No	6B, 6C, 6D, 8A, 8B
Au/Lu	Inversion	729	No	7B
Bu/Cd	Deletion	644	Yes	6D
Du/Eu	Inversion	1268	No	7C
Ed/Ad	Cut-and-paste	1785	No	8B
Ed/Dd	Inversion	1139	No	7C
Eu/Ad	Cut-and-paste	1192	No	8A
Eu/Dd	Deletion	546	Yes	6C, 6D
Eu/Ld	Inversion	1165	No	7D
Eu/Ld_0	Inversion	1093	No	7D
Eu/Lu	Cut-and-paste	586	Yes	8B
Lu/Ed	Inversion/cut-and-paste	1179	No	7D, 8A
Lu_0/Ed	Inversion	1336	No	7D
Iu/SDd	Deletion	1432	No	Supp. 3B
SBd/Yd	Inversion	783	Yes	Supp. 4B
sorrsAu/ltrbint30r	Ll.LtrB insertion	1354	No	Supp. 5B, 5C
sorrsBu/ltrbint30r	Ll.LtrB insertion	1317	No	Supp. 5B, 5C
sorrsCu/ltrbint30r	Ll.LtrB insertion	1328	No	Supp. 5B, 5C
sorrsDu/ltrbint30r	Ll.LtrB insertion	1166	No	Supp. 5B, 5C
sorrsEu/ltrbint30r	Ll.LtrB insertion	1345	No	Supp. 5B, 5C
sorrsFu/ltrbint30r	Ll.LtrB insertion	1329	No	Supp. 5B, 5C
sorrsGu/ltrbint30r	Ll.LtrB insertion	1592	No	Supp. 5B, 5C
sorrsHu/ltrbint30r	Ll.LtrB insertion	1624	No	Supp. 5B, 5C
sorrsIu/ltrbint30r	Ll.LtrB insertion	1340	No	Supp. 5B, 5C

*If an inverted repeat is generated, the actual size will usually be significantly smaller than the expected size due to loss of most of the repeated sequences via homologous recombination.
